# Exploring causality in the association between gut microbiota and irritable bowel syndrome risk: a large Mendelian randomization study

**DOI:** 10.18632/aging.205771

**Published:** 2024-04-25

**Authors:** Jishi Zhang, Xinlin Shi, Yun Wang

**Affiliations:** 1 Department of General Surgery, Huangdao District People’s Hospital, Qingdao, Shandong, China; 2 Department of Hepatology/Infectious Diseases, Huangdao District People’s Hospital, Qingdao, Shandong, China

**Keywords:** Mendelian randomization, irritable bowel syndrome, gut microbiota, risk

## Abstract

Background: In the past, some observational studies have highlighted the correlation between gut microbiota and irritable bowel syndrome (IBS). However, it is still unknown if the composition of gut microbiota shows a causal effect on the risk of IBS.

Aim: To conduct Mendelian randomization (MR) analysis of the samples to study the probable causal relationship between the gut microbiota, their taxonomic groups, and the risk of IBS.

Materials and Methods: In this study, the summarized data regarding 211 gut microbiota and their IBS genome-wide association studies (GWAS) were collected from public databases. The causal estimates were determined using five MR techniques, where Inverse Variance Weighted (IVW) regression was employed as the major MR technique. Herein, MR-PRESSO and MR-Egger intercept tests were conducted to prevent horizontal pleiotropy. Cochran’s *Q* test was used to evaluate heterogeneity using the IVW and MR-Egger techniques.

Results: IVW results showed that gut microbes, belonging to Class *Gammaproteobacteria* (*P* = 0.04; OR = 1.45), Family XIII (*P* = 0.03; OR = 1.34), Family *Prevotellaceae* (*P* = 0.003; OR =1.24), and *Lachnospiraceae* UCG004 (*P* = 0.049; OR = 1.19) increased the risk of IBS, while *Alcaligenaceae* (*P* = 0.03; OR = 0.83, 95% CI: 0.69–0.98) and *Coprobacter* (*P* = 0.02; OR = 0.86, 95% CI: 0.76–0.98) decreased the risk of IBS.

Conclusions: This study presented novel insights that highlighted the causal relationship between gut microbiota and IBS, and offered new treatment strategies for preventing or treating IBS.

## INTRODUCTION

Irritable bowel syndrome (IBS) refers to a functional gastrointestinal disorder that is characterized by alterations in bowel habits (constipation, diarrhea, or both) and recurring abdominal pain, usually accompanied by bloating and discomfort. IBS can negatively affect the quality of a patient’s life, and hence, it is regarded as a healthcare issue that places a huge economic burden on the national healthcare system. The sum of IBS-related direct and indirect costs in Europe is about 8 billion euros, 10 billion USD in the United States, and about 123 billion yuan in China [1]. Although this subject has been extensively studied in the past, the underlying pathophysiology and etiology of IBS is unclear. Some of the mechanisms of pathogenesis that have been established in earlier studies include increased intestinal permeability, visceral hypersensitivity, changes in the immune system, emotional disorders, and impaired intestinal motility [2]. The gut microbiota is involved in the pathogenesis of IBS, and its composition constitutes a complex and dynamic ecosystem including thousands of bacterial and microbial species that mainly inhabit the distal small intestine and colon [3]. Changes in the composition of the gut microbiota could lead to changes in intestinal motility, permeability, food processing, and visceral perception, ultimately resulting in the development of IBS-related symptoms [4]. In the past few years, several researchers have compared the differences in the gut microbiota and α-diversity between the healthy control individuals and patients with IBS. Their results showed that the α-diversity of fecal samples from IBS patients was lower than that presented by the healthy controls. The healthy controls and IBS patients also exhibited significant differences in the abundance and proportion of the gut microbes, and the changes in the specific microbial groups could cause microbial dysbiosis, which is a probable feature of IBS [5]. Furthermore, the increased risk of developing IBS following infectious enteritis and/or overuse of antibiotics also supports the hypothesis that gut microbiota dysbiosis is a factor that can lead to the onset of IBS symptoms [6, 7]. However, the results presented by the observational studies do not establish the causal link between gut microbiota and IBS. Confounding and reverse causation cannot be ruled out in observational studies owing to the lack of randomization of the exposure factors. Traditional randomized clinical controlled studies are methodologically appropriate but ethically problematic. Mendelian randomization (MR) is a technique that infers causation between the exposure factors and outcomes by using genetic variation as an instrumental variable for the exposure. The effect of confounding factors is significantly controlled since the genetic variations follow Mendelian's law and are distributed randomly within the populations [8]. As far as we know, this is the first study that employed the MR technique to assess the causal effect of gut microbes on the risk of IBS. Therefore, this study aimed to assess the causal correlation between the gut microbiota and the development of IBS by carrying out an MR analysis of all samples.

## MATERIALS AND METHODS

### Study design

**Figure 1:**
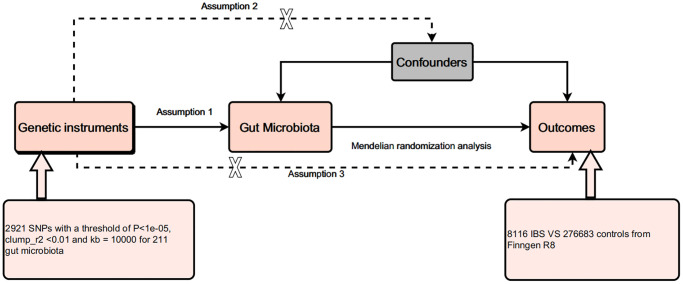
Design of this study.

MR is a method based on genetic information that aims to assess causality. Three key assumptions are required for conducting Mendelian randomization analysis: (1) The relevance assumption: the instrumental variable (usually single-nucleotide polymorphisms, SNPs, associated with a specific gene) should be significantly associated with the exposure variable (i.e., the independent variable in the study). This means that the selected genetic variant does indeed affect the exposure variable, making it an effective instrumental variable. (2) The independence assumption: the instrumental variable should not be related to any possible confounding factors. In other words, genetic variation should only affect the outcome variable (i.e., the dependent variable in the study) through the exposure variable, not through other pathways. This can be achieved by ensuring that the instrumental variable is unrelated to known and unknown confounding factors. (3) The exclusion restriction assumption: the instrumental variable should only affect the outcome variable using the exposure variable, not via another pathway. This means that there are no other pathways that affect the outcome variable, i.e., no horizontal pleiotropy. Horizontal pleiotropy refers to a genetic variant that simultaneously affects multiple phenotypes that are not related to the research purpose. Following the principles of MR, we conducted a causal analysis between the exposure factor, i.e., 211 gut microbiota, and the outcome factor, i.e., the onset of IBS. Figure 1 presents the flowchart of all experiments conducted in this study.

### Data source

The GWAS data regarding the gut microbiota were acquired from the Mibiogen Consortium, which included the abundance of 211 gut microbiota at five different levels (i.e., phylum, class, order, family, and genus) in the intestines of 18,340 participants of European ancestry. In this study, the composition of the gut microbes was analyzed using three different variable regions of the 16S rRNA gene (V4, V3-V4, and V1-V2), while the microbial quantitative trait loci (mbQTL) mapping technique was utilized for identifying the genetic variations affecting the relative abundance of the microbial groups [9]. The GWAS data on IBS were obtained from a study published by Wu et al. (2021), which included 28,518 IBS patients and 426,803 controls of European ancestry [10]. All data utilized in the study were acquired from publicly available databases and published studies, which have received ethical approval, so no further ethical approval was required.

### Instrumental variable selection

For gut microbiota, we selected SNPs with *P* < 1 × 10^−5^ as the threshold. Herein, the threshold was set to r^2^ <0.01 and Kb >10,000 for eliminating the linkage disequilibrium between SNPs. Palindromic SNPs would also be removed from the selection of instrumental variables. F-statistics were used for estimating the strength of all selected SNPs in describing the phenotype variation using the following formula: F = β²/SE². F >10 indicates that the selected SNP can significantly reduce potential bias, whereas F ≤ 10 indicates that the SNP is a weak instrumental variable [11]. Since IBS is influenced by factors such as psychological factors, diet, and intestinal infections, we used PhenoScanner2 to remove SNPs related to these confounding factors.

### Statistical analysis

In this study, five MR techniques were used for examining the causal effect of exposure on the outcomes, wherein the inverse variance weighting (IVW) technique was used as the primary MR technique. The supplementary techniques used in this study included MR-Egger, Simple mode, Weighted median, and Weighted mode techniques. This technique used MR-Egger, MR Pleiotropy RESidual Sum, and Outlier (MR-PRESSO) tests to check for effect heterogeneity, where *P* > 0.05 showed no effect heterogeneity. In the Cochrane Q statistic, MR-Egger and IVW methods were used for heterogeneity analysis, where *P* > 0.05 indicated no heterogeneity. Furthermore, the “Leave-one-out” sensitivity analysis was used to imply that the individual SNPs do not affect the causal effect of the exposure on the outcomes. All statistical analyses were performed using R software tools such as “TwoSampleMR”, “devtools”, “LDlinkR”, and “MRPRESSO”, and *P* < 0.05 was deemed as statistically significant.

### Data availability

The data used to support the findings of this study are included within the article.

## RESULTS

### Selection of instrumental variables

A total of 2921 SNPs were obtained from 211 gut microbiota, including 230 SNPs from 16 class-level gut microbes, 125 SNPs from 9 phylum-level gut microbes, 287 SNPs from 20 order-level gut microbes, 465 SNPs from 34 family-level gut microbes, and 1814 SNPs from 138 genus-level gut microbes. The F-value distribution ranged from 16.9 to 88.4, indicating that the presence of weak instrumental variables was unlikely (Supplementary Table 1). Additionally, no SNPs related to confounding factors were found through PhenoScanner2.

### MR analysis

**Figure 2:**
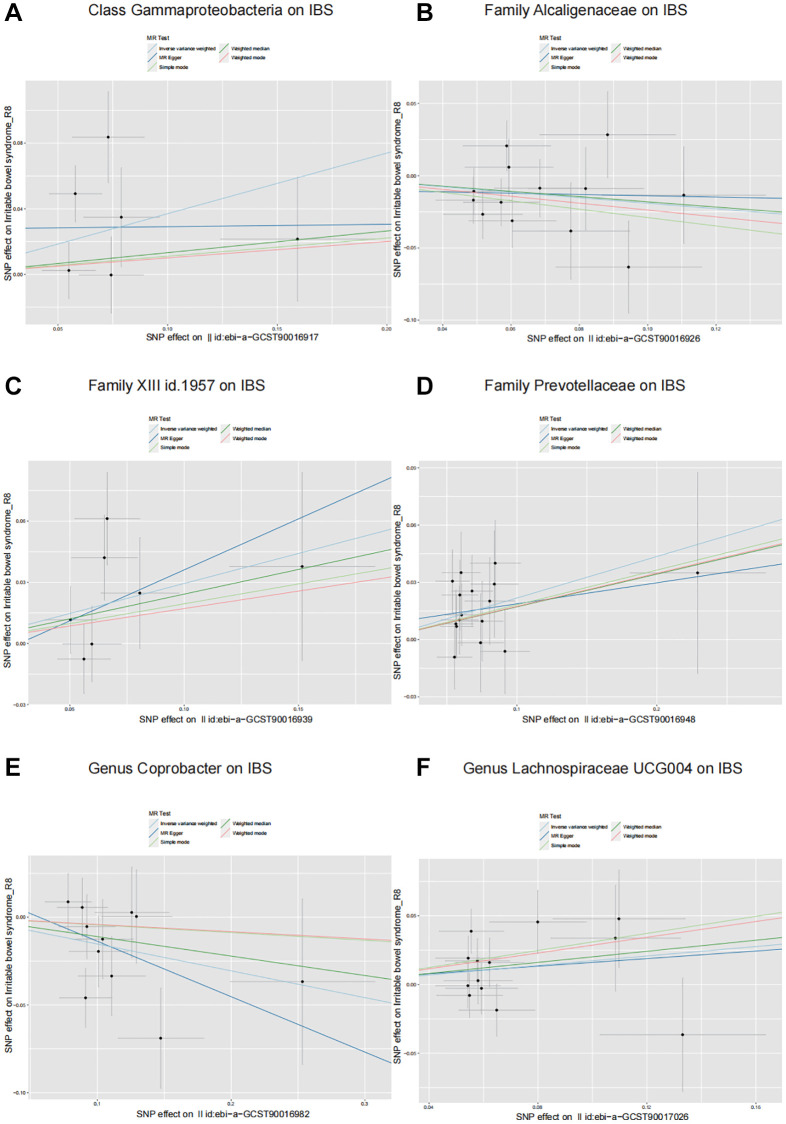
**Scatter plots illustrating the causal effects of six identified gut microbiota on IBS.** (**A**) Class *Gammaproteobacteria* on IBS. (**B**) Family *Alcaligenaceae* on IBS. (**C**) Family XIII on IBS. (**D**) Family *Prevotellaceae* on IBS. (**E**) Genus *Coprobacter* on IBS. (**F**) Genus *Lachnospiraceae* UCG004 on IBS.

The Class *Gammaproteobacteria*, represented by 6 SNPs, showed that for every unit increase in its abundance, the incidence of IBS increased by 1.45 times (*P* = 0.04; OR = 1.45, 95% CI: 1.02–2.06) according to the IVW results (Table 1 and Figure 2). The Family XIII, represented by 7 SNPs, showed that for every unit increase in its abundance, the incidence of IBS increased by 1.34 times (*P* = 0.03; OR = 1.34, 95% CI: 1.03–1.76). The Family *Prevotellaceae*, represented by 16 SNPs, showed that for every unit increase in its abundance, the incidence of IBS increased by 1.24 times (*P* = 0.003; OR = 1.24, 95% CI: 1.08–1.43) (Table 1 and Figure 2). The Family *Alcaligenaceae*, represented by 13 SNPs, showed that for every unit increase in its abundance, the incidence of IBS decreased by 0.83 times (*P* = 0.03; OR = 0.83, 95% CI: 0.69–0.98) (Table 1 and Figure 2). The Genus *Lachnospiraceae* UCG004, represented by 13 SNPs, showed that for every unit increase in its abundance, the incidence of IBS increased by 1.19 times (*P* = 0.049; OR = 1.19, 95% CI: 1.00–1.41) (Table 1 and Figure 2). The Genus *Coprobacter*, represented by 11 SNPs, showed that for every unit increase in its abundance, the incidence of IBS decreased by 0.86 times (*P* = 0.02; OR = 0.86, 95% CI: 0.76–0.98) (Table 1 and Figure 2). In these results, the OR values obtained by the MR-Egger, Weighted median, Simple mode, and Weighted mode algorithms were consistent with the IVW results, further confirming the reliability of the IVW results (Table 1). Supplementary Table 2 shows the original MR analysis results. No other gut microbiota was found to be causally related to IBS (Supplementary Table 3).

**Table 1:** Five MR methods of the causal relationships between 6 identified gut microbiota and IBS.

**Bacterial traits**		**SNP**	**Method**	**Beta (95% CI)**	**SE**	**OR (95% CI)**	***P***
Class	Gammaproteobacteria	6	MR Egger	0.01 (−1.12–1.15)	0.58	1.01 (0.33–3.17)	0.98
			Weighted median	0.13 (−0.23–0.49)	0.18	1.14 (0.79–1.65)	0.47
			IVW	0.37 (0.02–0.72)	0.18	1.45 (1.02–2.06)	0.04
			Simple mode	0.11 (−0.39–0.61)	0.26	1.12 (0.67–1.86)	0.69
			Weighted mode	0.10 (−0.32–0.52)	0.21	1.11 (0.77–1.59)	0.66
Family	Alcaligenaceae	13	MR Egger	−0.04 (−0.83–0.74)	0.40	0.96 (0.44–2.10)	0.91
			Weighted median	−0.18 (−0.42–0.06)	0.12	0.84 (0.66–1.05)	0.15
			IVW	−0.19 (−0.37–0.02)	0.09	0.83 (0.69–0.98)	0.03
			Simple mode	−0.29 (−0.71–0.13)	0.21	0.75 (0.49–1.14)	0.20
			Weighted mode	−0.24 (−0.64–0.16)	0.20	0.79 (0.53–1.17)	0.27
	Family XIII	7	MR Egger	0.50 (−0.57–1.57)	0.55	1.65 (0.56–4.82)	0.40
			Weighted median	0.24 (−0.09–0.57)	0.17	1.27 (0.91–1.78)	0.15
			IVW	0.29 (0.03–0.56)	0.14	1.34 (1.03–1.76)	0.03
			Simple mode	0.19 (−0.33–0.72)	0.27	1.21 (0.74–1.99)	0.47
			Weighted mode	0.17 (−0.27–0.61)	0.23	1.19 (0.74–1.91)	0.51
	Prevotellaceae	16	MR Egger	0.11 (−0.40–0.62)	0.26	1.12 (0.67–1.86)	0.68
			Weighted median	0.17 (−0.02–0.36)	0.10	1.19 (0.99–1.43)	0.07
			IVW	0.22 (0.08–0.36)	0.07	1.24 (1.08–1.43)	0.003
			Simple mode	0.18 (−0.14–0.51)	0.16	1.20 (0.86–1.67)	0.30
			Weighted mode	0.17 (−0.14–0.49)	0.16	1.19 (0.88–1.61)	0.28
Genus	Coprobacter	11	MR Egger	−0.32 (−0.81–0.19)	0.26	0.73 (0.44–1.20)	0.248
			Weighted median	−0.11 (−0.28–0.06)	0.09	0.90 (0.76–1.06)	0.19
			IVW	−0.15 (−0.28–0.03)	0.06	0.86 (0.76–0.98)	0.02
			Simple mode	−0.04 (−0.33–0.24)	0.15	0.96 (0.74–1.24)	0.75
			Weighted mode	−0.04 (−0.31–0.23)	0.14	0.96 (0.74–1.25)	0.76
	Lachnospiraceae UCG004	13	MR Egger	0.14 (−0.64–0.91)	0.40	1.15 (0.53–2.50)	0.73
			Weighted median	0.20 (−0.03–0.43)	0.12	1.22 (0.97–1.54)	0.09
			IVW	0.17 (0.00–0.35)	0.09	1.19 (1.00–1.41)	0.049
			Simple mode	0.31 (−0.12–0.74)	0.22	1.36 (0.88–2.11)	0.19
			Weighted mode	0.29 (−0.14–0.71)	0.22	1.33 (0.87–2.04)	0.21

### Sensitivity analysis

In this study, we performed several sensitivity analyses to check whether the results obtained by IVW methods were robust. The heterogeneity test did not reveal any heterogeneity in the results (*P* < 0.05). The MR-Egger regression intercept showed no significant deviation from zero, while all *p*-values of the global test in MR-PRESSO were observed to be more than 0.05, indicating no horizontal pleiotropy and no potential outliers among the IVs (Table 2). Additionally, the Leave-one-out results showed that MR analysis was not affected by individual SNPs (Supplementary Figure 1).

**Table 2:** Tests for heterogeneity and horizontal pleiotropy.

**Bacterial traits**		**Heterogeneity test**				**Pleiotropy test**			
		**IVW**		**MR-Egger**		**MR-Egger intercept**			**MR-PRESSO**
		**Q-statistics**	***P***	**Q-statistics**	***P***	**Estimate**	**SE**	***P***	**Global test *P***
Class	Gammaproteobacteria	10.34	0.07	9.35	0.05	0.03	0.04	0.56	0.1
Family	Alcaligenaceae	11.75	0.47	11.60	0.39	−0.01	0.03	0.72	0.47
	Family XIII	7.59	0.27	7.37	0.19	−0.01	0.04	0.71	0.31
	Prevotellaceae	8.01	0.92	7.82	0.90	0.01	0.02	0.67	0.93
Genus	Coprobacter	11.33	0.33	10.81	0.29	0.02	0.03	0.53	0.35
	Lachnospiraceae UCG004	13.47	0.34	13.46	0.26	0.002	0.03	0.93	0.37

## DISCUSSION

As a common functional gastrointestinal disorder, IBS has a global prevalence of 3–5%, with a significant geographical variation. It is estimated to have a prevalence rate of 10 to 25% in the United States, with South America showing the highest incidence rate (17–21%), while South Asia showed the lowest incidence rate (7–9%). The incidence rates in the Middle East and Africa are 5.6%. Although IBS does not pose a threat to life, it can cause a considerable degree of discomfort and pain [12, 13]. IBS is manifested in the form of different gastrointestinal symptoms, which also significantly affect the emotional health and well-being of the individuals. Most IBS patients display extra-intestinal manifestations, including clinically important psychiatric disorders [14]. IBS is often associated with higher levels of stress, decreased quality of life, and impaired work efficiency [15]. The human microbiome is composed of trillions of microorganisms, most of which coexist in the gut. The large and complex microbial community is 100 times greater than the total number of human cells. The gut microbiota undergoes constant changes throughout a person's lifetime due to various external and internal factors, and a series of reports have shown that alterations in microbial diversity and abundance are related to the pathogenesis of IBS [16]. Due to the heterogeneity and unclear etiology of IBS, it is difficult to identify specific biomarkers and therapeutic targets for IBS. The high diversity of the human gut microbiota also makes it difficult to determine clear beneficial or harmful microbial characteristics in the case of IBS [17]. The expansion of the microbial genome database has helped in understanding the involvement of the microbiome in various intestinal diseases. This study aimed to investigate the correlation between gut microbiota and IBS risk through a GWAS-based sample Mendelian randomization study. Gut microbiota has been considered a risk factor for gastrointestinal diseases. However, the causal association between IBS and gut microbiota remains uncertain. In this study, five different estimation methods (IVW, Simple mode, Weighted median, MR-Egger, and Weighted mode) were used for MR analysis. Our results showed the presence of a causal relationship between the risk of IBS and gut microbiota. The IVW method indicated that Class *Gammaproteobacteria* (*P* = 0.04; OR = 1.45), Family XIII (*P* = 0.03; OR = 1.34), Family *Prevotellaceae* (*P* = 0.003; OR = 1.24), and *Lachnospiraceae* UCG004 (*P* = 0.049; OR = 1.19) increased the risk of IBS, while *Alcaligenaceae* (*P* = 0.03; OR = 0.83, 95% CI: 0.69–0.98) and *Coprobacter* (*P* = 0.02; OR = 0.86, 95% CI: 0.76–0.98) decreased the risk of IBS. Sensitivity analysis techniques are required to assess the validity of the conclusions derived from the MR studies since they were more prone to bias from pleiotropy (correlation of the genetic variation with multiple variables). The weighted median technique was implemented for eliminating pleiotropy, as it generates valid estimates even if 50% of the SNPs were ineffective instruments [18]. The results of the Weighted median showed similar results to the IVW estimates, which increased the confidence level of these associations. Here, the MR-Egger regression technique was used to provide tests for unbalanced pleiotropy and causal estimates of the exposure on outcome [19]. The findings revealed that the intercept of the MR-Egger regression showed no significant deviation from 0, where the *p*-values of the MR-PRESSO global test were recorded to be >0.05, which indicated the absence of horizontal pleiotropy, while no IVs were described as potential outliers.

The results in this study showed that members of Class *Gammaproteobacteria*, Family XIII, Family *Prevotellaceae*, and *Lachnospiraceae* UCG004 could be regarded as important risk factors for IBS, while *Alcaligenaceae* and *Coprobacter* were seen to be protective factors for IBS. The elevated abundance of *Gammaproteobacteria* in the mucosa of IBS patients is regarded as a potential biomarker for inflammatory bowel disease [20]. In addition, literature reports have shown that persistent intestinal functional disorders after gastroenteritis are associated with a significant abundance of *Gammaproteobacteria* sp. The changes in the gut microbiota are related to diet, where higher fiber intake is associated with lower levels of *Gammaproteobacteria* sp [21]. The changes in the abundance of Family XIII and other gut microbiota can be regarded as indicators of childhood obesity and related cardiovascular damage [22]. Family XIII, as an inflammation-related clade, could be the primary driving factor of cancer-related fatigue in the gut-brain axis [23]. *Prevotellaceae* sp. is associated with a few human diseases like chronic periodontitis and inflammatory bowel disease [24, 25]. In an animal study, *Prevotellaceae* was found to be the main representative of chronic inflammation-related intestinal microbiota in mice [26]. Studies have pointed out that the IBS patients showed a higher proportion of *Prevotellaceae* in their duodenal mucosa than that noted in the healthy controls. In terms of the pathological physiology of human diseases, mucosal-related bacteria seem to be more important than luminal bacteria [27]. Depression and anxiety are common comorbid symptoms of IBD. *Lachnospiraceae* and *Prevotellaceae* display a higher predictive value for anxiety in Crohn's disease and ulcerative colitis [28]. A few studies have used the 16S rRNA gene sequencing technique, and their findings suggested a significant increase in the abundance of *Lachnospiraceae* sp. in diarrhea-predominant IBS patients [29]. A few protective factors are also beneficial to human health. Some of the representative members from the *Coprobacter* genus were isolated from the healthy human feces samples, however, none of the studies have highlighted their role in any intestinal pathology [30]. Recent studies highlighted a decreased abundance of *Coprobacter* sp. in patients with ulcerative colitis [31]. An MR analysis suggested that *Alcaligenaceae* sp. can reduce the risk of chronic kidney disease [32]. As a cellulose-degrading bacteria, *Alcaligenaceae* sp. indirectly promotes cellulose digestion and is enriched and actively expressed in the intestine microbiota of folivorous primates. Functional analysis shows that polysaccharide biosynthesis and metabolic pathways are significantly active [33].

The gut microbiota plays important roles in vitamin synthesis, metabolism of dietary compounds, maintaining the integrity of the intestinal epithelial barrier, regulating immune responses, and protection against intestinal pathogens [2]. The pathogenesis of IBS involves the interaction between the host and gut microbiota, which leads to the synthesis of several metabolites such as short-chain fatty acids, neurotransmitters, bile acids, and other signaling factors. The complex relationship between gut microbiota and the gut-brain axis shows a bidirectional link between IBS and psychosocial disorders [34]. Our findings are significant since they support the regulation of gut microbiota as an important intervention for the prevention and management of IBS. Compared to other risk factors of IBS (such as genetic susceptibility, food intolerance, visceral hypersensitivity, alteration of the gut-brain axis, motility disorder, immune dysfunction, etc.), the gut microbiota is changeable and can be easily regulated by improving the patient’s diet and supplementing probiotics. Therefore, if gut microbiota does indeed increase the risk of developing or exacerbating IBS, it becomes a hopeful target for controlling IBS [35, 36]. In summary, the results of the MR analysis present strong evidence highlighting the causal correlation between gut microbiota and the risk of IBS.

This study has a few advantages as it is a leading study where we have conducted a large-scale MR analysis between IBS and gut microbiota. Liu et al. also conducted a two-sample Mendelian randomization analysis to evaluate the relationship between intestinal microbiota and irritable bowel syndrome, but we believe that our study still has greater significance [37]. The population data we selected for our study differs from that of Liu et al. Liu used the GWAS summary data of Eijsbouts et al., which included 53,400 cases and 433,201 controls of European ancestry [38]. Our study selected Wu et al.’s GWAS summary data, which included 28,518 IBS patients and 426,803 controls of European ancestry [10]. The birth of a scientific conclusion relies on data from multiple sources, and different data sources may lead to varying conclusions. In Liu et al.’s study, they found a causal relationship between IBS and 10 types of intestinal bacteria, whereas our study found a causal relationship with 6 types of intestinal bacteria. Our findings contradict those of Liu et al. There are several reasons for this discrepancy, which could be attributed to the heterogeneity of intestinal flora among different populations. This emphasizes the importance of investigating the causal relationship between gut bacteria and IBS using diverse datasets. Additionally, our methodology differs from Liu et al. In Mendelian randomization analysis, it is crucial that the selected SNPs of intestinal bacteria are not associated with confounders of the outcome. This ensures that bias is not introduced in the MR analysis results. Possible risk factors for IBS include psychological factors, diet, and intestinal infections. In our study, we aimed to identify SNPs associated with these risk factors using the PhenoScanner2 database, which was not utilized in the study conducted by Liu et al. Although we did not discover any SNPs related to confounding factors among the candidate SNPs in the PhenoScanner2 database, our research methodology was more rigorous, taking into account the aforementioned factors. The MR technique has been implemented in the study design, which considered the confounding factors and reverse causality. In this study, the experimental data were derived from the published GWAS studies with genetic variations and large sample sizes. However, a few limitations remain. Firstly, although this study investigated the proportion of the gut microbiota and implied that the IBS patients showed a higher diversity and heterogeneity of gut microbiota, the data used in this study were still limited. Secondly, this study only analyzed the European population without sex differentiation, which indicates that when the findings were generalized to other ethnicities’ populations, we need to be cautious while presenting the conclusions. Finally, although the MR method could provide novel insights into the causal relationship between the exposure and outcome features, the degree of association may not be accurately estimated. Hence, additional research is required for validating the results of this study.

## Supplementary Materials

Supplementary Figure 1

Supplementary Table 1

Supplementary Table 2

Supplementary Table 3
