# Supplementary material for: Exploring causality in the association between gut microbiota and irritable bowel syndrome risk: a large Mendelian randomization study
Source: Aging (Albany NY). 2024 Apr 25;16(8):7448–59. doi: 10.18632/aging.205771 (PMC11087118; doi:10.18632/aging.205771)
Supplement: Supplementary Figure 1 [file aging-16-205771-s001.pdf]

SUPPLEMENTARY FIGURE

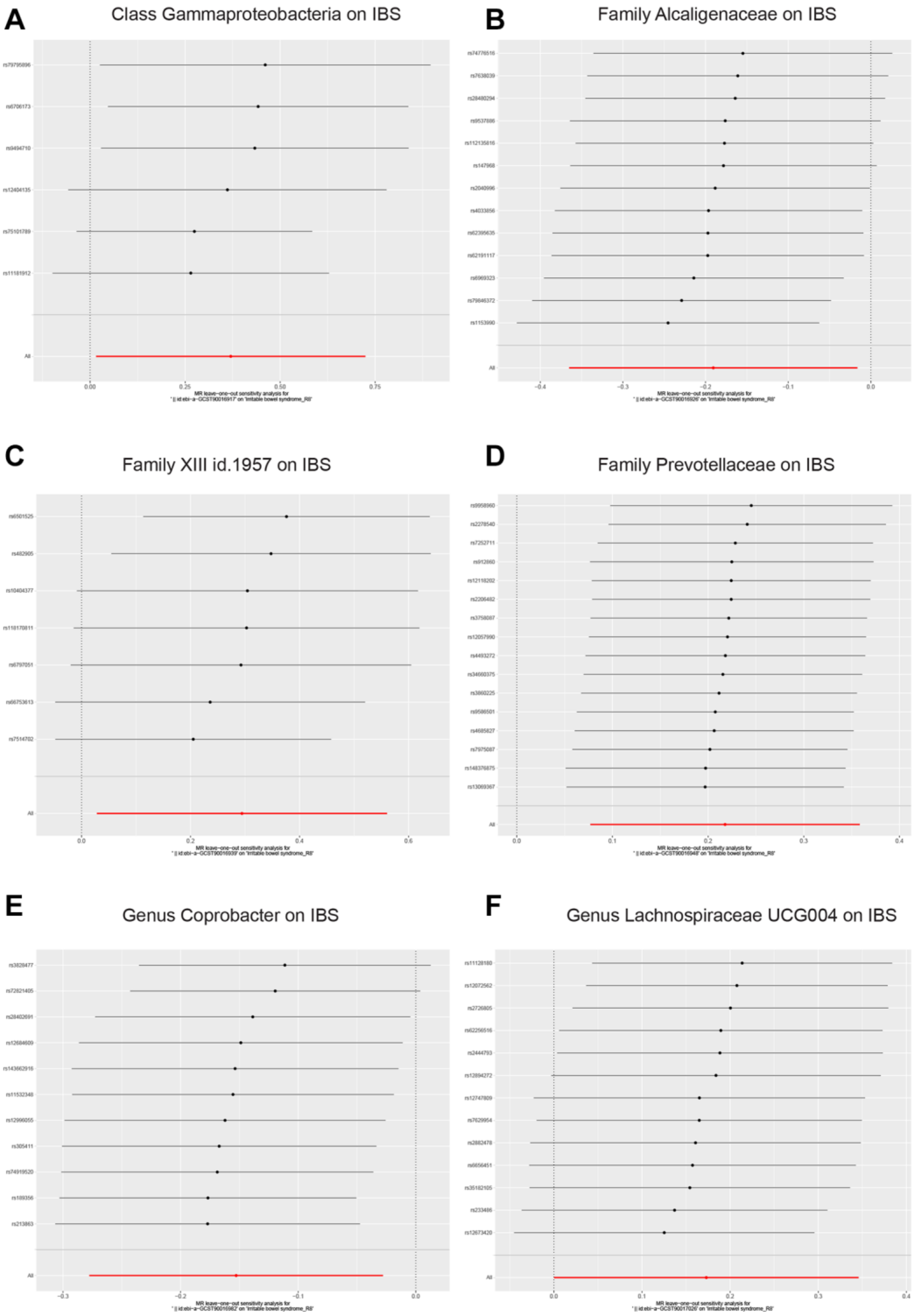

Supplementary Figure 1. Forest plots illustrate the results of sensitivity analyses with leave-one-out for the six associations that have been identified. (**A**) Class *Gammaproteobacteria* on IBS. (**B**) Family *Alcaligenaceae* on IBS. (**C**) Family XIII on IBS. (**D**) Family *Prevotellaceae* on IBS. (**E**) Genus *Coprobacter* on IBS. (**F**) Genus *Lachnospiraceae* UCG004 on IBS.
